# Learning Curve, Survival Curve

**DOI:** 10.1200/JGO.19.00303

**Published:** 2020-04-13

**Authors:** Reena George, Thotampuri Shanthi Prasoona, Ramu Kandasamy, Shakila Murali, Roja Rekha, Thenmozhi Mani

**Affiliations:** ^1^ Palliative Care Unit, Christian Medical College, Vellore, Tamil Nadu, India; ^2^ Department of Continuing Medical Education, Christian Medical College, Vellore, Tamil Nadu, India; ^3^ Department of Biostatistics, Christian Medical College, Vellore, Tamil Nadu, India

## THE FIRST 10 YEARS: UNSEEN SUFFERING

They came to us from the slums of India’s metropolis and its rural hinterland—poverty-stricken women with cervical cancer. Their names would be put on the 4-month-long waiting list for radiotherapy. The women waited, but the cancer did not. Somewhere in the world, one woman was dying of cervical cancer every 2 minutes, but, in the hospital, for 16 hours on a working day, the radiotherapy machine lay unused, the gamma rays of its steadily decaying radioactive cobalt source spent into the lead shield.

Patient attrition was inevitable. Some died, some were too ill to return, some families gave up. After 4 months, the surviving fraction of patients received 33 fractions of external radiotherapy without brachytherapy. Disease recurrence was common, and the smell of vaginal discharge often announced treatment failure before the pelvic examination confirmed it. The women rarely returned to hospital. The trajectory and symptoms of incurable cervical cancer were not described in our textbooks; we did not have to answer questions about such clinical scenarios in our exit examinations.

I passed my examinations, completed my residency, and moved to my old medical school in Vellore, South India. The telecobalt machine worked two shifts, so we could start radiotherapy without delay. We used radioactive sources in our hand-held manual after-loading brachytherapy applicator. We followed the common sense precautions of radiation protection: distance, time, and barrier. A miniscule risk to our health could not be more important than a near-definite death sentence to a patient who did not get brachytherapy.

The nurse in the outpatient clinic gave pre-radiotherapy vaginal douches to a long line of women each morning to wash out necrotic vaginal discharge. Later in the day, she would do dressings for mutilating head and neck cancers, fungating breast primaries, and tumors with maggots. Even through the difficult smells and sights, she met her patients with kind words and a smile. I marveled at her ability to do that because, despite my efforts, I retched easily in response to smell.

The women with recurrent cervical cancer would try to make us, female doctors, understand the severity of their pain. It was much worse than labor pain they said, and it had gone on for months, not hours. We did not have oral morphine in those days. When our limited repertoire of analgesics failed and pain made it difficult to walk, the women stopped coming. Uncontrolled pain, uncontrolled emotions, and uncontrolled smell were three big barriers that made me hesitant to get involved in palliative care. It took years to muster up the courage to venture into the field.^1^

## THE NEXT 10 YEARS: UNMANAGEABLE SYMPTOMS

After a few years of palliative care training in the United Kingdom, I returned to the hospital in Vellore and gradually moved from radiation oncology to full-time palliative care. A small team came together. Once we obtained oral morphine, we could reduce pain, but smell remained a problem. When a malodorous patient came to our clinic, we tried to see her without delay, to make it easier for ourselves and for others in the clinic. We wondered how the patient had managed to travel to hospital in a crowded public bus. As we began to go on home visits, we saw what happened to women who did not return to hospital—flies congregating around the deathbed, maggots, bedclothes soaked with urine and stool from vesicovaginal and rectovaginal fistulae.

K. had a rectovaginal fistula and was admitted into the radiation oncology ward in 2004. She was too ill to undergo diversion surgery. The smell hit us as soon as we entered the corridor. Despite our medical and nursing efforts, the stench persisted, and other patients complained. K. went back to the hut she shared with her daughter’s family. When her daughter came to collect pain medicines from our clinic, she would tell us about the smell that pervaded life in their two-room hut. The smell made it difficult for her to cook and difficult for the children to eat. We could imagine the mess but could do nothing to resolve the prob-lem. Finally, K. took matters into her own hands. When the family had gone to church, bedridden K, with a hemoglobin of < 5 g, managed to raise herself, tie a noose with a sari*,* and put an end to the agony of her caregivers. Fistula and smell had precipitated suicide.

Smell also affected others attending the clinic. When women with fistulae soiled the benches in the waiting room, other patients stopped coming, and their pain, too, went unaddressed. Cervical carcinoma was the second-leading cause of cancer deaths among women, but, unlike breast cancer, there was little research into specific palliative needs. Therefore, we published a special issue on advanced cervical cancer in the *Indian Journal of Palliative Care*.^2-4^

Although ours was not high-level evidence, it was evident that cervical cancer was difficult to palliate. We shared our different symptom management strategies and homely remedies.^5^ The books recommended vaginal douches with metronidazole solutions, and some even recommended instilling diluted vinegar or soda-bicarbonate solutions into the vagina.^6^ By the mid-2000s we were buying kilograms of metronidazole powder, which our pharmacist compounded into solutions for douches and dressings. But compliance was poor, because it is not easy to put the nozzle of a douche can into a vagina mutilated by tumor or fistula. Gradually, we started using oral, rather than topical metronidazole. In palliative oncology and in palliative care, a treatment that a patient is able to sustain long term is often more effective than something that is likely to be discontinued because of cost, toxicity, or complexity.

Faced with such suffering, demands we could not meet, unmitigated responsibilities, after 13 years in palliative care, I took a sabbatical year.

## THE NEXT 5 YEARS: UNDERSTANDING MECHANISMS

Sometimes it is easier to notice a change when one has been away for a while. A few months after I returned to work, I remarked, “We are not having as many ‘smelly days’ in the clinic. Could it be because we are prescribing regular oral metronidazole?”

“Yes, smell is less of a problem,” our social worker confirmed. “I used to purchase room-freshening sprays every month. It's been a long time since we had to buy one.”

“We rarely see maggots, secretions are less,” our nurse reported.

Prompted by these simple observations, we decided to do a retrospective study. We found that, with increasing use of regular oral metronidazole, the proportion of smelly visits had come down. We read up, trying to understand the chemistry of malodor, and learned that volatile fatty acids were not the primary cause of smelly fungating tumors. The main culprit was di-methyl trisulfide.^7^ Prior experience with noxious hydrogen sulfide in school chemistry laboratories convinced us that di-methyl trisulfide was, indeed, the villain.

But, on reflection, we had to admit that di-methyl trisulfide was not a villain, it was a very essential part of our ecosystem. Its aroma was a magnet for carrion flies.^8^ And the maggot larvae of those flies cleaned up decomposing corpses and put nitrogenous nutrients back into nature’s food chain. The problem was when maggots were born within the necrotic tumors of living human beings. To reduce smell, flies, and maggots, we had to stem the production of di-methyl sulfide at its source—within the cancer, with timely metronidazole.

The WHO analgesic ladder had taught us to titrate painkillers to mild, moderate, or severe cancer pain,^9^ so we devised a “SNIFFF ladder”^9a^ for smell. Nil, faint, foul, and forbidding degrees of smell were managed with their respective doses of metronidazole.^10^ When families coming from other towns thanked us for getting rid of long-uncontrolled malodor, we realized that one of our most difficult symptoms had become manageable with maintenance metronidazole.

## UNSCHOOLED TEACHERS

Muniamma received maintenance metronidazole. Widowed, illiterate, living on 30 dollars a month, she was also the sole caregiver for an adult daughter with special needs. Rani was 32 and had a mental age of about 6, after a childhood CNS infection had left her with 2 emaciated flailing lower limbs, slurred speech, and a warm smile for familiar faces.

As we entered the 10-by-12–foot hut, I was amazed that Muniamma had managed to complete 5 weeks of external radiotherapy and 2 of her 3 brachytherapy applications. She would have had to leave Rani alone, walk across a riverbed, change 2 buses, and travel 40 kilometers each way, morning and evening. That took considerable courage and self-discipline. Many others with cervical cancer drop out of treatment.

We listened to Muniamma’s symptoms, helped Rani to crawl out of the hut, and did a pelvic and rectal examination with Muniamma lying on the floor of her hut. We explained medications and asked if there were any questions. Many of our families inquire about dietary recommendations, but Muniamma and Rani did not have the luxury of culinary choices. Rice, subsidized by the government, was boiling in a pot on the fire. A few dried red chilies sat on the plate. The chilies would serve as the sole vegetable, condiment, and curry for the day.

We began to make inquiries about potential long-stay homes for Rani. The wardens wanted to know if Rani was self-caring during her menstrual periods. ‘Yes,’ Rani told us. Walking on 2 upper limbs and dragging her lower limbs, she would go down to the small river 300 meters away to do her laundry. Like many poor women, Muniamma and Rani could not afford sanitary pads. Lack of access to diapers and to transport are barriers to accessing cervical cancer care.^11,12^

Muniamma, however, continued to come regularly to collect her palliative medicines. We would also send her with some cooking oil and lentils to supplement the diet of rice and red chilies. As the tumor progressed and the rectal lumen became narrower, I suggested an elective colostomy. Muniamma backed out, saying that Rani would be frightened by the stoma. It was a difficult decision. I was very worried. How would Muniamma travel to hospital if she developed total obstruction or a rectovaginal fistula? What would Rani do? Each month, I examined the vagina and the rectal lumen and listened carefully to the history. Was the pelvic pain due to cancer or obstruction? The former required an increase in morphine, the latter warranted a diversion surgery.

A few months later, Muniamma developed a small defect between the vagina and the rectum. To my great surprise, despite pelvic disease, the rectovaginal fistula closed within a few weeks. Gradually, I began to wonder if the fistula had healed because Muniamma was getting low-dose metronidazole every day. Like an abscess, anaerobic secretions within a hypoxic cervical cancer could open into the rectum or bladder to create a fistula. And necrotic discharge would keep that fistula open. Without persistent discharge, fibrosis or progressive tumor might close the defect.

Although I had not expected Muniamma to survive long with a bulky pelvic recurrence and hydronephrosis, she lived with a nearly occluded rectum for over 2 years. Finally, 3 years after she was referred to palliative care, and with near-total obstruction, Muniamma underwent a colostomy. Not long after, she also developed a urinary fistula and was confined to the floor of her hut.

Rani took over the household responsibilities. Her older sister who worked in another district came intermittently to collect medications and help at home. Our team cut old absorbent cotton saris and T-shirts into towels, bed linen, and diapers and taught Rani how to use them. Equipped with these makeshift swaddling clothes, Rani mothered her dying mother in her final months. Muniamma died in her hut, 44 months after recurrence was diagnosed.

Muniamma’s healed fistula prompted us to look again at patients who had received maintenance metronidazole. We found that maintenance metronidazole was associated with a significant 3-fold reduction in vesicovaginal and rectovaginal fistulae.^9a,13^ When fistulae were prevented or postponed, women like Muniamma remained ambulant and self-caring for longer. Perhaps that was why we also found an association with a longer postrecurrence survival. Retrospective data have many limitations, and we hope maintenance metronidazole will be studied prospectively in other settings. Metronidazole is on the WHO essential drugs list and is accessible even to women who might never reach a radiotherapy department.^14^ It costs us less than 1 dollar to prescribe maintenance metronidazole for a month in our country.

## UNSUNG LEARNERS

Some time after Muniamma’s death, we visited the bereaved Rani. She had not wanted to move to a long-stay home or to her sister’s house. She lived alone and greeted us with her big smile. The bare little hut was neat and clean. Rani told us that, each Friday, she took out her possessions and washed out her dwelling. It is so much easier, I thought, to spring clean—a house or a life—when one has little baggage.

But how did Rani know that it was Friday? When all the neighbors washed out their houses, Rani knew that it was cleaning day. And when the postman came down the village street, it was time to put the rice to cook. Although Rani could not read clocks or calendars, she had created a rhythm for her life.

## CONNECTING THE DOTS

Like Rani, we too had learned from simple observations. Often, we learned the most important lessons from those who were the least confident in their own ability. Our impoverished patients have taught us about courage, faith, and what really matters in life.^15^ But this time we had found a nugget of science hidden within a deep necrotic tumor—something that we had missed for many years even though it was, literally, under our nose.

For me, it has been 28-year journey with advanced cervical cancer. As I look back to connect the dots—events and losses, memories and milestones—I am grateful that a learning curve has slowly matured into a fistula-free survival curve.^13^

## References

[1] George R Empty hands J Palliat Care 18 200 201 2002 12418372

[2] Muckaden MA Marathe M Tulshan R et al Psychosocial issues faced by women with incurable cervical cancer in India: How can we help? Indian J Palliat Care 11 94 2005

[3] George R Doubly disadvantaged: Dying of cervical cancer Indian J Palliat Care 11 62 2005

[4] Casilda S Krishnaswamy M Wound care in resource-poor settings Indian J Palliat Care 11 105 2005

[5] George R Srinivasan V From kitchen bench to bedside Ann Intern Med 165 223 224 2016 27479223 10.7326/M15-2352

[6] Palliative Care for Women With Cervical Cancer A Kenya field manual https://path.azureedge.net/media/documents/RH_palliative_care_guide.pdf

[7] Shirasu M Nagai S Hayashi R et al Dimethyl trisulfide as a characteristic odor associated with fungating cancer wounds Biosci Biotechnol Biochem 73 2117 2120 2009 19734656 10.1271/bbb.90229

[8] Johnson SD Jürgens A Convergent evolution of carrion and faecal scent mimicry in fly-pollinated angiosperm flowers and a stinkhorn fungus S Afr J Bot 76 796 807 2010

[9] WHO WHO’s cancer pain ladder for adults https://www.who.int/cancer/palliative/painladder/en/

[9a] George R, Prasoona TS, Kandasamy R, et al: Regular low-dose oral metronidazole is associated with fewer vesicovaginal and rectovaginal fistulae in recurrent cervical cancer: Results from a 10-year retrospective cohort. J Glob Oncol doi: 10.1200/JGO.19.00206 10.1200/JGO.19.00206 PMC6733185 31479340

[10] George R Prasoona TS Kandasamy R et al Improving malodour management in advanced cancer: A 10-year retrospective study of topical, oral and maintenance metronidazole BMJ Support Palliat Care 7 286 291 2017 10.1136/bmjspcare-2016-001166 28174164

[11] Bates MJ Mijoya A A review of patients with advanced cervical cancer presenting to palliative care services at Queen Elizabeth Central Hospital in Blantyre, Malawi Malawi Med J 27 93 95 2015 26715953 10.4314/mmj.v27i3.4 PMC4688869

[12] Tapera O Dreyer G Kadzatsa W et al Determinants of access and utilization of cervical cancer treatment and palliative care services in Harare, Zimbabwe https://ssrn.com/abstract=3314449 10.1186/s12889-019-7355-3 PMC6664562 31357977

[13] George R Prasoona TS Kandasamy R et al Regular low-dose oral metronidazole is associated with fewer vesicovaginal and rectovaginal fistulae in recurrent cervical cancer: Results from a ten-year retrospective cohort J Glob Oncol 5 1 10 2019 10.1200/JGO.19.00206 PMC6733185 31479340

[14] Mocanu V Dang JT Ladak F et al Antibiotic use in prevention of anal fistulas following incision and drainage of anorectal abscesses: A systematic review and meta-analysis Am J Surg 217 910 917 2019 30773213 10.1016/j.amjsurg.2019.01.015

[15] George R Life’s lessons lost...and learned J Clin Oncol 28 1806 1807 2010 20124160 10.1200/JCO.2009.27.0413

